# Anthocyanins as Adjuvant Treatment for Non-alcoholic Fatty Liver Disease: A Systematic Review and Meta-Analysis

**DOI:** 10.7759/cureus.63445

**Published:** 2024-06-29

**Authors:** Naveed N Khan, Elaf J Zurayyir, Maryam Y Almuslem, Riyadh Alshamrani, Razan A Alamri, Ghalia Hasan T Sulaimani, Mamdouh Hussein T Sulimani, Maryam Salah F Albalawi, Rawan M Alzehair Alqahani, Eman M Alanazi, Huda H Aljawi, Jawad A Alsuliman

**Affiliations:** 1 Internal Medicine, King Salman Armed Forces Hospital, Tabuk, SAU; 2 Medicine, Jazan University, Jazan, SAU; 3 Medicine, King Faisal University, Al-Ahsa, SAU; 4 Family Medicine, Bisha University, Bisha, SAU; 5 Medicine, Taif University, Taif, SAU; 6 Medicine, Ibn Sina National College for Medical Studies, Jeddah, SAU; 7 Internal Medicine, King Saud University, Riyadh, SAU; 8 General Medicine, Alexandria University, Riyadh, SAU; 9 Internal Medicine, King Faisal Specialist Hospital & Research Centre, Makkah, SAU; 10 Critical Care, Prince Saud Bin Jalawy Hospital, Al-Ahsa, SAU

**Keywords:** non-alcoholic fatty liver disease, metabolic-associated fatty liver, aspartate aminotransferases, alanine transaminase, anthocyanins

## Abstract

Recent studies suggest a role for anthocyanins in the treatment of non-alcoholic fatty liver disease (NAFLD). The purpose of the present review was to assess the effect of anthocyanins as an adjuvant treatment in patients with NAFLD. The literature search was conducted on MEDLINE/PubMed, the Cochrane Central Register of Controlled Trials (CENTRAL), the Web of Science, and Scopus without language or time limits up to March 27, 2024. The primary outcomes included the severity of liver fibrosis and the level of liver transaminases. Secondary outcomes included obesity and lipid profile assessments. Standardized mean differences (SMDs) with 95% CIs were calculated for numerical outcomes. Five studies were included. The pooled effect sizes showed lower levels of liver fibrosis and liver transaminases in the anthocyanin group, but the difference was nonsignificant and small in size. The same result was obtained with anthropometric measurements of total cholesterol, low-density lipoprotein, and serum triglycerides, where effect sizes ranged from negligible to medium in magnitude but were all nonsignificant. The anthocyanin group showed a significantly lower body fat percentage (SMD = -0.41 (95%CI: -0.76; -0.06), P = 0.021). Currently, no evidence is available on the efficacy of anthocyanins in improving liver fibrosis or dyslipidemia in patients with NAFLD. There is limited evidence that anthocyanins can lower body fat percentages, but the effect was not reflected in the pooled results of other obesity indices. The few available clinical trials showed several limitations and variations regarding the doses of anthocyanins. Future clinical trials should avoid the limitations of the current studies and provide evidence supporting or refuting the use of anthocyanins in NAFLD patients.

## Introduction and background

Non-alcoholic fatty liver disease (NAFLD) is a condition in which fat droplets (particularly triglycerides) accumulate in hepatocytes and constitute more than 5% of liver weight in the absence of chronic alcohol consumption [1]. Worldwide, NAFLD is considered the most prevalent chronic liver disease [2,3]. Globally, the prevalence of NAFLD is 25% in the adult population but rises in diabetic patients to reach at least 50% [4].

Recently, the nomenclature of NAFLD was changed to metabolic-associated fatty liver disease (MAFLD) [5]. The diagnostic criteria of MAFLD include the presence of hepatic steatosis besides at least one of the following three features: (1) overweight based on BMI; (2) type 2 diabetes mellitus (T2DM); and (3) lean or normal weight with evidence of metabolic dysregulation [6].

The condition can present as simple steatosis or become complicated by chronic inflammation and tissue damage (steatohepatitis). Fibrosis of varying degrees often develops in cases of steatohepatitis. Steatohepatitis by itself is not life-threatening, but the patients have a higher risk of developing liver cirrhosis and hepatocellular carcinoma [2,3]. In addition, several morbid conditions have been associated with NAFLD, including cardiovascular disease, which is the main cause of death in those patients [7,8], metabolic syndrome [9], and hypertension [10].

The exact mechanisms involved in the pathogenesis of NAFLD are not yet clear. The proposed hypotheses include steatosis caused by insulin resistance, inflammation, and oxidative stress resulting in progression [11,12], and impaired proliferation of progenitors of liver cells [5]. Several factors contribute to the development and progression of NAFLD, including obesity, dyslipidemia, inflammation, and insulin resistance [13]. Therefore, modifiable lifestyle factors (e.g., weight loss and a healthy diet) play an important role in the management of NAFLD [14,15], as no definitive drug therapy has been established [16]. Research endeavors to find new, effective treatments for NAFLD patients.

Among the widely investigated treatments for NAFLD, plant-based substances have been a focus of research. Various bioactive compounds were tested in clinical trials, such as anthocyanins in recent years [17-21], and curcumin [22].

Anthocyanins are water-soluble pigments that belong to the flavonoid family. Anthocyanins give rise to the reddish-orange to bluish-purple color that is seen in various fruits and vegetables. Previous studies found that anthocyanins possess antioxidant, anti-inflammatory, and anti-atherosclerotic properties and thus can decrease the risk of obesity-related morbidities [23]. The present meta-analysis aims to assess the effect of anthocyanins as an adjuvant treatment in patients with NAFLD.

## Review

Methods

The meta-analysis followed the principles of the Cochrane Handbook for Systematic Reviews of Interventions, version 6. The methods and results were reported in accordance with the Preferred Reporting Items for Systematic reviews and Meta-Analyses (PRISMA) guidelines [24].

Eligibility criteria for the included studies

Types of Studies

This meta-analysis included only clinical trials, without restrictions in time or language.

Participants

Studies were included if patients were diagnosed with NAFLD.

Interventions

Eligible studies included a direct comparison between anthocyanins and a placebo or standard treatment. The included studies must provide a quantification of the administered anthocyanins.

Exclusion Criteria

The following records were excluded: conference abstracts, duplicate reports, observational studies, review articles, editorials, clinical guidelines, non-human research (in vitro or animal studies), and studies lacking a comparator.

Search Strategy

The search was conducted in the electronic databases of MEDLINE/PubMed, Cochrane Central Register of Controlled Trials (CENTRAL), Web of Science, and Scopus. No filters were used (except search words that were used to exclude animal studies; see Appendix A). The search included all studies from inception until March 27, 2024. The search terms included “anthocyanin*” AND “non-alcoholic fatty liver”. The search terms used for each database are outlined in Appendix A.

Selection of Studies

Two independent reviewers conducted the search strategy, screened the retrieved results, and assessed the full text of potentially eligible studies. Disagreements between the two reviewers were settled by consulting the third reviewer.

Data Extraction

The following data were extracted from the included studies: (a) the study place (country), design, time span, eligibility criteria, sample size, and the duration of follow-up; (b) patients’ age and sex; (c) the details of the study arms; and (d) the outcomes: fibrosis score, anthropometric measurements (body weight, BMI, body fat, and waist/hip circumference), and the lipid profile (total cholesterol, high-density lipoprotein (HDL), low-density lipoprotein (LDL), and serum triglycerides).

Measured Outcomes

Primary outcomes: Comparison of the severity of fibrosis (fibrosis score) and liver transaminases between the two groups at the end of the study.

Secondary outcomes: Comparison of the indices of obesity (anthropometric measurements) and lipid profile (total cholesterol, HDL, LDL, and serum triglycerides) between the two groups at the end of the study.

Assessment of the Risk of Bias (ROB) in Included Studies

The ROB in the included studies was assessed using the ROB2 tool for clinical trials [25]. The tool consists of five domains that assess the randomization process, deviations from the assigned treatment, missing data, measurement of the outcome, and selective reporting of the outcomes and results. In addition, an overall ROB can be assessed from the five domains.

Data Synthesis

The R Statistical Software version 4.3.2 [26], with the packages “meta” version 7.0.0 [27] and “dmetar” version 0.1.0 [28], was used for the calculation of the standardized effect sizes, pooling the results, and creating forest plots. We also created a narrative synthesis table [29] to report the direction of effect for each outcome as well as summarize the results of heterogeneity testing and the pooled estimates. All outcomes were numerical and were presented using the standardized mean difference (SMD) by subtracting the mean for the anthocyanin group from the mean for the control group and then dividing the result by the pooled standard deviation. We selected SMD over mean difference as the studies differed in the units used for reporting the laboratory analyses. Significant heterogeneity was detected at a P-value <0.1 from the Cochrane chi-square test and an I² index ≥50%. If heterogeneity was nonsignificant, the fixed effects model was used for pooling the estimates. Alternatively, the random effects model was used if heterogeneity was significant [30]. A P-value <0.05 was used for interpreting the comparisons between the two arms. The effect size for SMDs was classified as large (SMD ≥0.8), medium (≥0.5), small (≥0.2), and negligible (<0.2) [31]. No testing was performed for publication bias as the number of included studies was less than 10.

Results

Results of the Literature Search and Study Selection

**Figure 1:**
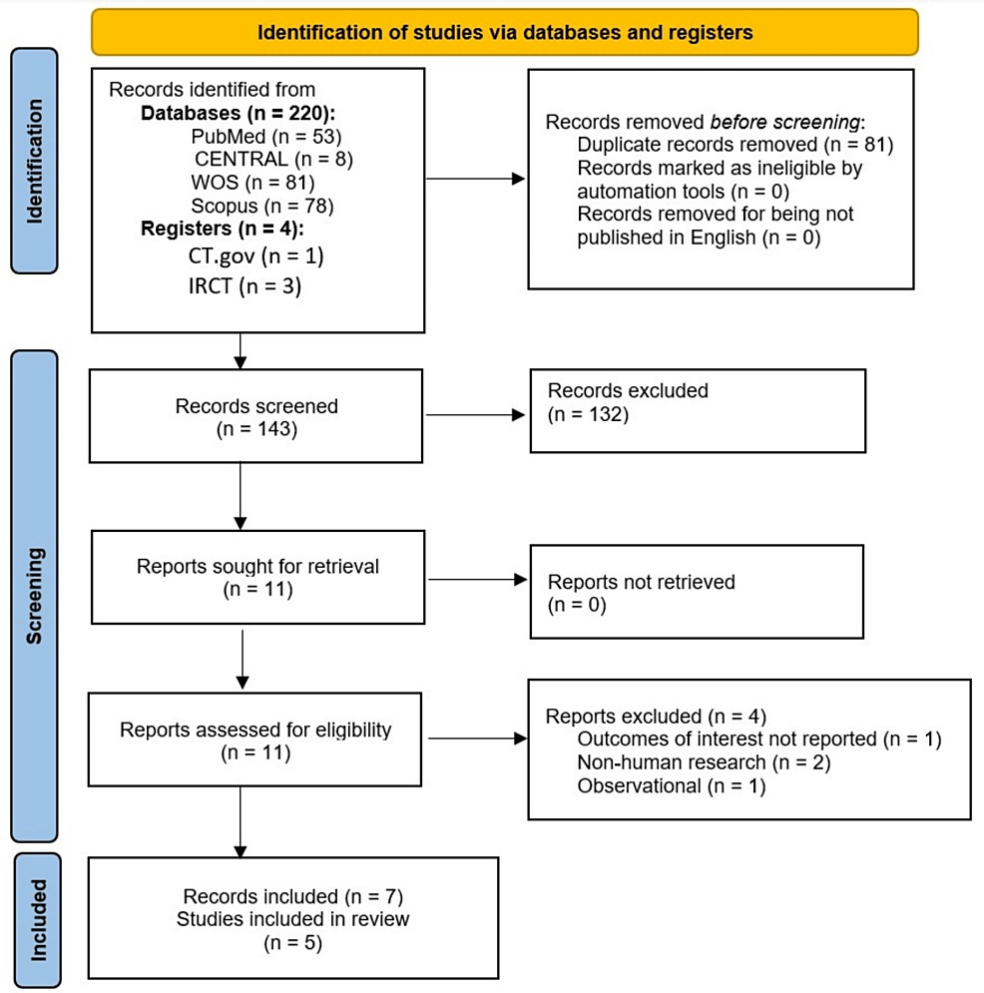
PRISMA flow chart diagram for the results of the literature search and study selection PRISMA: Preferred Reporting Items for Systematic reviews and Meta-Analyses

The online literature search yielded 220 records from the searched databases and four records from the trial registries. Eighty-one records were duplicates and were removed. The remaining 143 records underwent screening of the titles and abstracts, leading to the exclusion of 132 records. The full texts of the remaining 11 records were retrieved and evaluated for the eligibility criteria of the current meta-analysis. We excluded four records that did not meet the eligibility criteria, leaving seven records for inclusion [17-21,32,33]. Out of the eligible seven records, two records [20,33] were found to be reports from two other studies [17,18]; thus, the included studies were five (Figure 1).

Basic Characteristics and Assessment of the ROB of the Included Studies

The basic characteristics of the included studies: Tables 1-3 summarize the basic characteristics and interventions of the five included studies. The design was a randomized controlled trial in all studies. The studies were conducted in Taiwan [32], China [17,33], Iran [18-20], and Turkey [21]. The duration of the studies and follow-up were 12 weeks in three studies [17,18,20,32,33] and eight weeks in the other two (Table 1) [19,21].

**Table 1:** Characteristics of the included studies (n = 5) CM: Cornus mas; RCT: randomized controlled trial

Study	Study design	Country	Time span	Sample size CM: control	Follow-up (weeks)
Chang et al. [32]	RCT	Taiwan	July 2007 to June 2009	19:17	12
Zhang et al. [17]	RCT	China	June 2013 to October 2013	37:37:00	12
Sangsefidi et al. [18] and Yarhosseini et al. [20]	RCT	Iran	May 2019 to August 2019	25:25:00	12
Mojiri-Forushani et al. [19]	RCT	Iran	January 2021 to March 2022	45:45:00	8
Bayram et al. [21]	RCT	Turkey	June 2021 to May 2022	50:50:00	8

**Table 2:** Patients’ characteristics and eligibility criteria of the included studies (n = 5) ALT: alanine transaminase; AM: anthocyanin; C: control; CVD: cardiovascular disease; CM: Cornus mas; F: female; M: male; NAFLD: non-alcoholic fatty liver disease; NSAIDs: nonsteroidal anti-inflammatory drugs; OTC: over the counter; T2DM: type 2 diabetes mellitus

Study	Age (years)	Gender (M/F)	Inclusion criteria	Exclusion criteria
Chang et al. [32]	CM: 37.32 ± 8.61; C: 38.59 ± 10.51	CM: 12/7; C: 9/8	Males or nonpregnant females aged 18-65 with a BMI ≥27, fatty liver, and not under a course of treatment	Drinking habit (≥20 g alcohol daily), ALT 3-fold higher or bilirubin >2 mg/dL, kidney dysfunction, CVD, endocrine or severe systemic disturbance, mental disorder, or taking any OTC or prescribed medication and nutraceuticals
Zhang et al. [17]	AC: 44.9 ± 7.5; C: 46.9 ± 7.7	AC: 19/18; C: 20/17	NAFLD, age from 25 to 65 years, recruited at the Shaoguan Railway Hospital, fatty liver, plasma ALT >30 U/L in men and 19 U/L in women	Excessive alcohol consumption (ethanol >140 g/wk for men and >70 g/wk for women), cirrhosis, viral hepatitis, CVD, cancer, NSAIDs, corticosteroids, prescriptive medicines affecting liver function, lipid, or glucose metabolism
Sangsefidi et al. [18] and Yarhosseini et al. [20]	CM: 41.48 ± 9.53; C: 42.68 ± 9.96	CM: 12/13; C: 11/14	NAFLD; fatty liver grades 1, 2, or 3; 25-65 years of age; ALT levels >30 U/L in men and >19 U/L in women; living in Yazd city	Alcohol consumption; viral hepatitis; cancer; Wilson disease; T2DM; CVD; mental diseases; pregnancy; lactation; adherence to a special diet 1 month before the study; taking corticosteroids; NSAIDs; hypoglycemic drugs; tamoxifen; Na valproate; methotrexate; amiodarone; anti-retroviral agents for HIV; probiotics; antioxidant and anti-inflammatory supplements e.g., vitamins D or E or omega 3; resveratrol during one month before the study
Mojiri-Forushani et al. [19]	AC: 37.71 ± 9.39; C: 36.04 ± 9.40	AC: 26/19; C: 26/19	NAFLD patients, 20-50 years old, BMI 30–40 kg/m^2^	Alcohol use, lactation or pregnancy, athlete and menopausal, inflammatory conditions (e.g., hypertension and infection), family history of hyperlipidemia, CVD, renal, lung, or liver disease, biliary disease, cancer, liver transplantation, autoimmune disease, injuries and burns during the research, surgery in the last three months, taking medications (e.g., insulin sensitivity enhancers, antihypertensives, statins, hepatotoxic drugs, estrogens, contraceptive pills, or antioxidants) in the last two months
Bayram et al. [21]	CM: 43.90 ± 10.44; C: 43.40 ± 12.46	CM: 10/12; C: 10/12	Aged 18-65 years; alcohol consumption in the past one year not >20 g/day for men and 10 g/day for women; diagnosis of steatosis; resident of the city of Istanbul	Hepatitis B or C; Wilson disease; hemochromatosis; Cushing syndrome; autoimmune liver disease; CVD; cancer; mental diseases; severe liver or kidney dysfunction; thyroid diseases; prolonged use of estrogen or regular consumption of drugs associated with fatty liver diseases (e.g., corticosteroid, methotrexate, tamoxifen, and amiodarone); pregnancy, breastfeeding, allergy to CM fruit

**Table 3:** Intervention and comparator arms in the included studies (n = 5) C: control; CM: Cornus mas; HSE: *Hibiscus sabdariffa *extract

Study	Intervention	Comparator
Chang et al. [32]	Two 450 mg HSE (2.5% anthocyanins) capsules after meals, three times a day (about 67.5 mg/day total anthocyanin)	Placebo capsules
Zhang et al. [17]	Two anthocyanin 80-mg capsules extracted from blueberry and black currant (320 mg/day total anthocyanin)	Placebo capsules
Sangsefidi et al. [18] Yarhosseini et al. [20]	20 ml/day CM extract (32 mg/day total anthocyanin)	20 ml/day of purified water and a red color such as carmoisine color
Mojiri-Forushani et al. [19]	One capsule per day of dried hydroalcoholic grape seed extract (38 mg anthocyanin)	Placebo capsules
Bayram et al. [21]	30 g/day lyophilized CM fruit powder (350 mg/day total anthocyanin)	No CM or diet therapy

The inclusion and exclusion criteria, as well as the sex distribution of the patients, varied slightly among the studies (Table 2).

The sources of anthocyanins included *Hibiscus sabdariffa *extract [32], blueberry and black currant extracts [17], Cornus mas (CM) extract [18,20,21]¸ and grape seed extract [19]. The total daily dose varied widely across the studies, with doses below 100 mg/day in three studies [18-20,32] and doses above 300 mg/day in two studies [17,21,33]. Placebo was provided in all studies except in one study where standard treatment for NAFLD was only provided (Table 3) [21].

The assessment of the ROB in the included studies: As regards the randomization process, all studies provided details about the generation of random numbers and allocation concealment, except the study by Chang et al., which showed some concerns [32]. As for the deviations from intended interventions, two studies showed high risk as not all participants completed the follow-up and intention-to-treat analysis was not used [21,32]. Missing outcome data represented a concern in four studies, with three of them having high ROB [17,18,20,32], and one study with some concerns [21]. The ROB was low regarding the measurement of outcomes, as blinding of outcome assessors was reported in most studies, and the recording of anthropometric measurements or laboratory analyses could hardly be affected by non-blinding. In addition, the risk of selective reporting of results was low in four studies and raised some concerns in one study only [32], as no protocol or prespecified analysis plan was available. The assessment of overall ROB in the ROB2 tool depends on the highest degree of risk observed within the study. Consequently, four studies had a high ROB [17,18,20,21,32,33]¸ while one study only had a low overall ROB (Table 4) [19].

**Table 4:** The ROB assessment for the included studies based on the ROB2 tool ROB: risk of bias

Study	Randomization process	Deviations from intended interventions	Missing outcome data	Measurement of the outcome	Selection of the reported result	Overall
Chang et al. [32]	Some concerns	High	High	Low	Some concerns	High
Zhang et al. [17]	Low	Low	High	Low	Low	High
Sangsefidi et al. [18] Yarhosseini et al. [20]	Low	Low	High	Low	Low	High
Mojiri-Forushani et al. [19]	Low	Low	Low	Low	Low	Low
Bayram et al. [21]	Low	High	Some concerns	Low	Low	High

Results of the meta-analysis

Fibrosis Score

**Figure 2:**
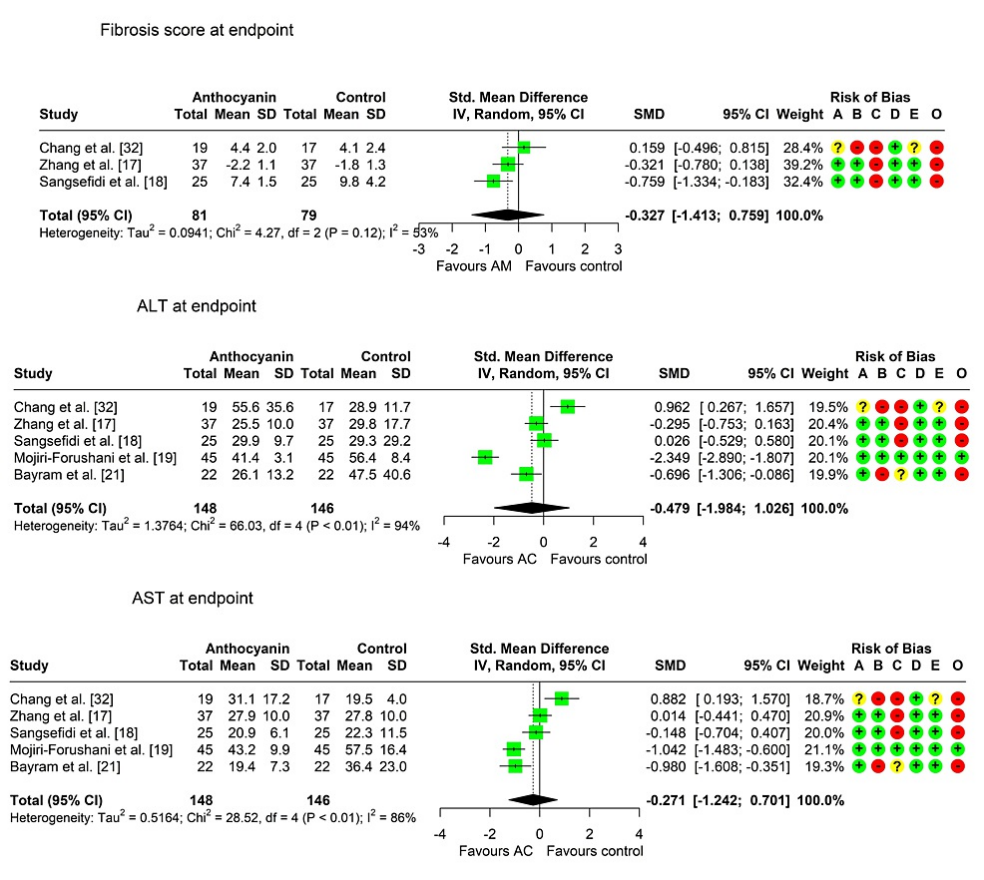
Forest plot showing pooling of the studies’ findings regarding the fibrosis score, ALT, and AST levels A: randomization process; B: deviations from intended interventions; C: missing outcome data; D: measurement of the outcome; E: selection of the reported result; O: overall risk of bias ALT: alanine transaminase; AST: aspartate transaminase; SMD: standardized mean difference

Three studies compared the fibrosis score between the two arms [17,18,32]. Two studies reported a lower score (positive effect) in the anthocyanin group at the end of the study compared to the control group [17,18], but the difference was statistically significant in one study only [18]. The third study reported a slightly higher average score in the anthocyanin group, but the difference was not statistically significant. There was considerable heterogeneity among the studies (Chi² = 4.27, P-value = 0.118, I² = 53.1%), so pooling of the results was achieved using the random effects model. The pooled SMD (95% CI) was -0.33 (-1.41, 0.76), P-value = 0.325, indicating a small, nonsignificant decrease in the fibrosis score in the anthocyanin group compared to the control group (Table 5, Figure 2).

**Table 5:** Summary of the main results of narrative synthesis and meta-analysis a: positive effect refers to a decrease in the intervention group while negative effect refers to an increase in the intervention group b: effect size for SMDs: large ≥0.8, medium ≥0.5, small ≥0.2, and negligible <0.2 *: significant ALT: alanine transaminase; AST: aspartate transaminase; HDL: high-density lipoprotein; LDL: low-density lipoprotein; SMD: standardized mean difference

Outcomes	Studies (participants) number for meta-analysis	Direction of effect a	Statistically significant effects of a	Effect estimate SMD/OR (95% CI), model, P	Interpreting effect size b	Heterogeneity
Fibrosis score	3 (160)	Positive: 2/3 [17,18]; Negative: 1/3 [32]	Positive: 1/3 [18]; Negative: 0/3	SMD = -0.33 [-1.41, 0.76], Random, P = 0.325	Small	Chi² = 4.27 (P = 0.118); I² = 53.1%
ALT	5 (294)	Positive: 3/5 [17,19,21]; Negative: 2/5 [18,32]	Positive: 3/5 [17,19,21]; Negative: 0/5	SMD = -0.48 [-1.98, 1.03], Random, P = 0.427	Small	Chi² = 66.03 (P < 0.001*); I² = 93.9%
AST	5 (294)	Positive: 3/5 [18,19,21]; Negative: 2/5 [17,32]	Positive: 2/5 [19,21]; Negative: 0/5	SMD = -0.27 [-1.24, 0.70], Random, P = 0.483	Small	Chi² = 28.52 (P < 0.001*); I² = 86%
Body weight	5 (294)	Positive: 4/5 [17,19-21]; Negative: 1/5 [32]	Positive: 0/5; Negative: 0/5	SMD = -0.09 [-0.32; 0.14], Fixed, P = 0.439	Small	Chi² = 1.88 (P = 0.758); I² = 0%
BMI	4 (244)	Positive: 3/4 [17,19,21]; Negative: 1/4 [32]	Positive: 1/4 [21]; Negative: 0/4	SMD = -0.20 [-0.45, 0.06], Fixed, P = 0.130	Small	Chi² = 2.17 (P = 0.538); I² = 0%
Body fat percentage	3 (130)	Positive: 3/3 [20,21,32]; Negative: 0/3	Positive: 3/3 [20,21,32]; Negative: 0/3	SMD = -0.41 [-0.76; -0.06], Fixed, P = 0.021*	Small	Chi² = 0.23 (P = 0.893); I² = 0%
Waist circumference	4 (204)	Positive: 2/4 [20,21]; Negative: 2/4 [17,32]	Positive: 1/4 [21]; Negative: 0/4	SMD = -0.10 [-0.37, 0.18], Fixed, P = 0.491	Negligible	Chi² = 2.22 (P = 0.529); I² = 0%
Hip circumference	4 (204)	Positive: 2/4 [20,21]; Negative: 2/4 [17,32]	Positive: 1/4 [20]; Negative: 0/4	SMD = -0.12 [-0.39; 0.16], Fixed, P = 0.409	Negligible	Chi² = 4.38 (P = 0.223); I² = 31.5%
Total cholesterol	4 (244)	Positive: 4/4 [17,19,21,32]; Negative: 0/4	Positive: 2/4 [19,21]; Negative: 0/4	SMD -0.50 [-1.58; 0.58], Random, P = 0.236	Small	Chi² = 19.79 (P < 0.001*); I² = 84.8%
HDL	4 (244)	Positive: 1/4 [32]; Negative: 3/4 [17,19,21]	Positive: 0/4; Negative: 0/4	SMD = 0.08 [-0.17; 0.34], Fixed, P = 0.516	Negligible	Chi² = 0.59 (P = 0.898); I² = 0%
LDL	4 (244)	Positive: 3/4 [17,19,21]; Negative: 1/4 [32]	Positive: 3/4 [17,19,21]; Negative: 0/4	SMD = -0.51 [-1.48, 0.47], Random, P = 0.197	Medium	Chi² = 15.77 (P = 0.001*); I² = 81%
Serum triglycerides	4 (244)	Positive: 4/4 [17,19,21,32]; Negative: 0/4	Positive: 3/4 [19,21,32]; Negative: 0/4	SMD = -0.59 [-1.41, 0.24], Random, P = 0.108	Medium	Chi² = 12.44 (P = 0.006*); I² = 75.9%

Alanine Transaminase (ALT)

All studies compared the ALT measurements between the two groups. Three studies reported a lower mean (positive effect) in the anthocyanin group compared to the control group [17,19,21], with a statistically significant difference. Two studies reported a higher ALT level in the anthocyanin group relative to the control, but the results were not significant [18,32]. Pooling of results was performed using the random effects model due to the marked heterogeneity among the studies (Chi² = 66.03, P-value < 0.001, I² = 93.9%). The pooled SMD (95% CI) was -0.48 (-1.98, 1.03), P = 0.427, indicating a small, nonsignificant decrease in ALT in the anthocyanin group compared to the control group (Table 5, Figure 2).

Aspartate Transaminase (AST)

All studies compared the AST measurements between the two groups. Three studies reported a lower mean (positive effect) in the anthocyanin group compared to the control group [18,19,21], with a statistically significant difference in two studies [19,21]. Meanwhile, two studies reported a higher AST level in the anthocyanin group, but the results were not significant [17,32]. Pooling of results was achieved using the random effects model due to the marked heterogeneity among the studies (Chi² = 28.52, P-value < 0.001, I² = 86%). The pooled SMD (95% CI) was -0.27 (-1.24, 0.70), P = 0.483, indicating a small, nonsignificant decrease in AST in the anthocyanin group compared to the control group (Table 5, Figure 2).

Body Weight

**Figure 3:**
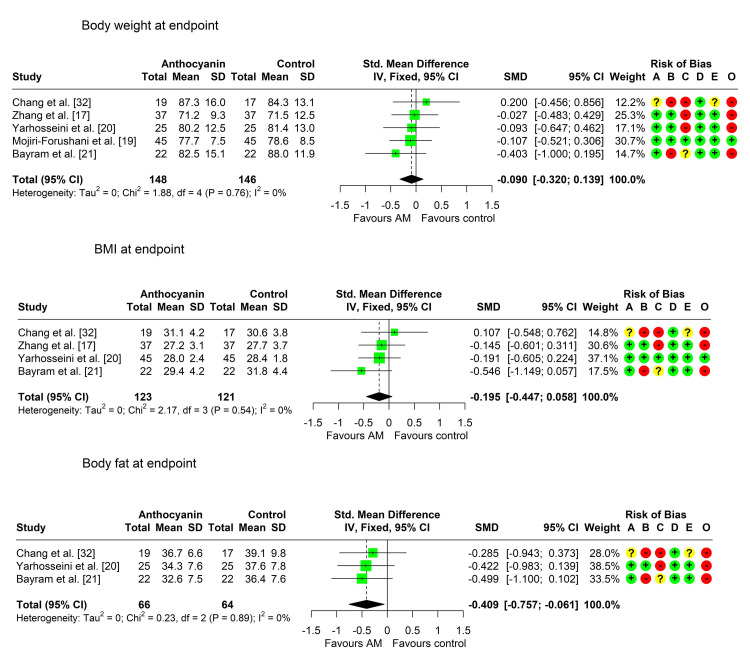
Forest plot showing pooling of the studies’ findings regarding body weight, BMI, and body fat percentage A: randomization process; B: deviations from intended interventions; C: missing outcome data; D: measurement of the outcome; E: selection of the reported result; O: overall risk of bias SMD: standardized mean difference

All studies recorded the difference in body weight between the two arms. Four studies reported a nonsignificantly lower mean body weight in the anthocyanin group compared to the control group [17,19-21], while one study reported a nonsignificant increase in the intervention group [32]. Heterogeneity testing was nonsignificant (Chi² = 1.88, P = 0.758, I² = 0%), so the fixed effects model was used. The pooled SMD (95% CI) was -0.09 (-0.32; 0.14), P = 0.439, indicating a small, nonsignificant decrease in body weight in the intervention group compared to the control (Table 5, Figure 3).

BMI

Four studies compared the BMI between the two arms [17,19,21,32]. Three studies reported a lower mean BMI in the anthocyanin group [17,19,21], reaching statistical significance in one study only [21]. One study reported a higher mean BMI in the anthocyanin group, but the result was nonsignificant [32]. Heterogeneity was not significant (Chi² = 2.17, p = 0.538, I² = 0%), so data were pooled using the fixed effects model. The pooled SMD (95% CI) was -0.20 (-0.45, 0.06), P = 0.130, indicating a small, nonsignificant decrease in BMI in the anthocyanin group (Table 5, Figure 3).

Body Fat Percentage

Three studies compared the percentage of body fat between the two arms [20,21,32], showing significantly lower mean values in the anthocyanin group than in the control group. Heterogeneity was not significant (Chi² 0.23, P = 0.893, I² = 0%), so a fixed effects model was used. The pooled SMD (95% CI) was -0.41 (-0.76, -0.06), P = 0.021, indicating a small, significant decrease in the percentage of body fat in the anthocyanin group relative to the control group (Table 5, Figure 3).

Waist Circumference

Four studies compared the waist circumference between the two arms [17,20,21,32]. Two studies reported a lower mean in the anthocyanin group compared to the control group [20,21], with a significant difference in one study [21]. The other two studies reported a nonsignificantly higher mean in the anthocyanin group [17,32]. Heterogeneity was not significant (Chi² = 2.22 (P = 0.529); I² = 0%), so pooling was performed using the fixed effects model. The pooled SMD (95% CI) was -0.10 (-0.37, 0.18), P = 0.491, indicating a negligible, nonsignificant decrease in waist circumference in the anthocyanin group relative to the control group (Table 5).

Hip Circumference

Four studies compared the waist circumference between the two arms [17,20,21,32]. Two studies reported a lower mean in the anthocyanin group compared to the control group [20,21], with a significant difference in one study [20]. The other two studies reported a nonsignificantly higher mean in the anthocyanin group [17,32]. Heterogeneity was not significant (Chi² 4.38, P = 0.223, I² = 31.5%), so pooling was performed using the fixed effects model. The pooled SMD (95% CI) was -0.12 (-0.39; 0.16), P = 0.409, indicating a negligible, nonsignificant decrease in waist circumference in the anthocyanin group relative to the control group (Table 5).

Total Cholesterol

**Figure 4:**
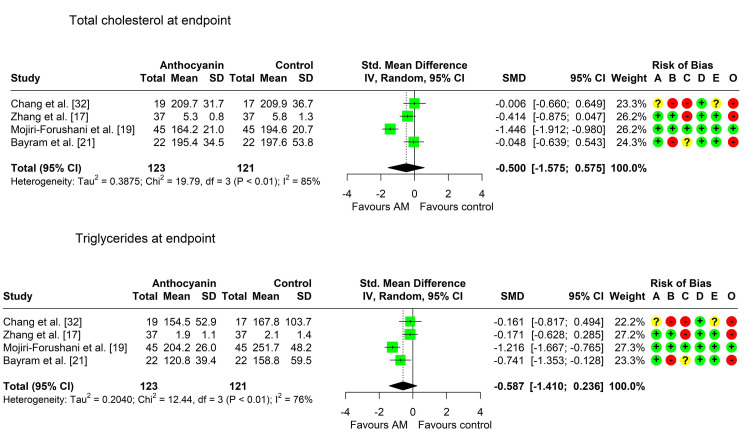
Forest plot showing pooling of the studies’ findings regarding serum levels of total cholesterol and triglycerides A: randomization process; B: deviations from intended interventions; C: missing outcome data; D: measurement of the outcome; E: selection of the reported result; O: overall risk of bias SMD: standardized mean difference

Four studies assessed the difference in total cholesterol levels between the two arms [17,19,21,32], all of them reporting a lower mean in the anthocyanin group. However, two studies only found a significant difference [19,21]. Heterogeneity was marked across the studies (Chi² 19.79, P < 0.001, I² = 84.8%), so we pooled the data using the random effects model. The pooled SMD (95% CI) was -0.50 (-1.58; 0.58), P = 0.236, indicating a small, nonsignificant decrease in the total cholesterol level in the anthocyanin group compared to the control group (Table 5, Figure 4).

HDL

Four studies assessed the difference in HDL levels between the two arms [32]. Increased HDL was found in three studies [17,19,21]. One study only reported a lower mean in the anthocyanin group [32] and the other three reported a higher mean, with no significant differences in any of the studies. Heterogeneity was nonsignificant across the studies (Chi² = 0.59, P = 0.898, I² = 0%), so we pooled the data using the fixed effects model. The pooled SMD (95% CI) was 0.08 (-0.17; 0.34), P = 0.516, indicating a negligible, nonsignificant increase in HDL level in the anthocyanin group compared to the control group (Table 5).

LDL

Four studies assessed the difference in LDL levels between the two arms [17,19,21,32]. Three studies reported a significantly lower mean in the anthocyanin group [17,19,21]. One study reported a nonsignificantly higher level in the anthocyanin group compared to the control group [32]. Heterogeneity was marked across the studies (Chi² = 15.77, P = 0.001, I² = 81%), so we pooled the data using the random effects model. The pooled SMD (95% CI) was -0.51 (-1.48, 0.47), P = 0.197, indicating a medium, nonsignificant decrease in the LDL level in the anthocyanin group compared to the control group (Table 5).

Serum Triglycerides

Four studies assessed the difference in serum triglycerides levels between the two arms [17,19,21,32], all of them reporting a lower level in the anthocyanin group, with a significant difference in the anthocyanin group in three of them [19,21,32]. Heterogeneity was marked across the studies (Chi² = 12.44 (P = 0.006*); I² = 75.9%), so we pooled the data using the random effects model. The pooled SMD (95% CI) was -0.59 (-1.41, 0.24), P = 0.108, indicating a medium, nonsignificant decrease in serum triglycerides level in the anthocyanin group compared to the control group (Table 5, Figure 4).

Sensitivity Analyses

Outlier and influence analysis as well as leave-one-out analysis were performed on the studied outcomes. Outlier studies were detected for ALT [19,32], AST [19,32], and total cholesterol [19]. The pooled estimates after the removal of outliers were SMD = -0.479 (-1.984; 1.026), random model, P = 0.427 for ALT; SMD = -0.271 (-1.242; 0.701), random model, P = 0.483 for AST; and SMD = -0.500 (-1.575; 0.575), random model, P = 0.236 for total cholesterol.

Discussion

Summary of the Main Findings

NAFLD represents the most common chronic liver disease worldwide and is associated with serious comorbidities and complications. Currently, no definitive pharmacotherapy exists for NAFLD, and treatment relies mainly on lifestyle modification [16]. Anthocyanins are promising plant-based interventions that have demonstrated positive effects in treating obesity and chronic inflammation [23,34]. The present meta-analysis aims to assess the effect of anthocyanins as an adjuvant treatment in patients with NAFLD. Seven records were retrieved, which originated from five studies.

In patients with NAFLD, liver transaminases may show any pattern, but ALT and/or AST levels are increased above the normal range in more than 70% of patients [35]. The pooled effect sizes in the current meta-analysis showed small, nonsignificantly lower levels of both ALT (SMD = -0.48 (95% CI: -1.98, 1.03), P = 0.427) and AST (SMD = -0.27 (95% CI: -1.24, 0.70), P = 0.483) in the anthocyanin group compared to the control group. This result coincides with the findings of Soltani et al., who reported that the intake of 300 mg of anthocyanins (derived from CM extract) for six weeks did not significantly decrease ALT and AST levels in patients with T2DM [36].

Research emphasizes that losing at least 3-5% of the body weight improves steatosis, and a loss of 7-10% improves inflammation, hepatocyte ballooning [37], and even fibrosis in non-alcoholic steatohepatitis [38]. Our results showed that pooled SMD suggested lower measurements of body weight, BMI, as well as waist and hip circumferences in the anthocyanins group compared to the control group at the end of the study; however, that decrease was nonsignificant and negligible to small in size. Meanwhile, the difference between the two groups regarding body fat percentage showed statistically significant decreased values in the anthocyanins group compared to the control, but the effect size was also small (SMD = -0.41 (95% CI: -0.76, -0.06), P = 0.021).

The results of clinical trials investigating the effect of anthocyanins on obesity in different populations are controversial [36,39,40]. A study by Lee et al. reported that, after 12 weeks of administering cranberry extract (a rich source of anthocyanins) to patients with T2DM, the change in waist circumference and BMI did not significantly differ from the control group [39]. In addition, Basu et al. assessed the effect of an eight-week blueberry intake on patients with metabolic syndrome and reported a lack of significant differences in weight and waist circumference between the intervention and control groups [41]. Likewise, Soltani et al. conducted a trial on T2DM patients and showed that the intake of 300 mg of anthocyanin resulted in a nonsignificant decrease in BMI compared to placebo [36]. On the other hand, Gholamrezayi et al. found that the administration of CM extract containing 900 mg anthocyanin for eight weeks in postmenopausal women resulted in a significant decrease in body weight, BMI, and waist [40]. The dosage of anthocyanins in the latter study was markedly higher than the studies included in our meta-analysis. A systematic review that included studies on obese subjects found that anthocyanin supplementation of 300 mg/day or less for four weeks was sufficient to reduce BMI and body weight [42].

It is possible that the improvement of obesity measurements requires higher doses of anthocyanins, but we were unable to assess this issue due to the small number of studies available for inclusion in our analysis. Future studies should evaluate the effect of different doses of anthocyanins in NAFLD patients on reducing obesity and establish the safety of high doses in those patients.

The proposed mechanisms for the effect of anthocyanins on weight loss include the alteration of mitogen-activated protein kinase (MAPK) and the NF-κB stress signaling pathway, leading to cytoprotective and anti-inflammatory effects. Another plausible mechanism is through the elevation of adiponectin levels and the decrease in leptin secretion and fat deposition. In addition, anthocyanins may act by inhibiting pancreatic lipase activity, which results in decreased intestinal fat absorption and consequently lessens visceral fat accumulation [43,44].

Another important outcome that we investigated in this meta-analysis was the potential effect of anthocyanin intake on the lipid profile of NAFLD patients. A strong association exists between NAFLD and dyslipidemia, as the prevalence of NAFLD in dyslipidemia subjects reaches 50%, and dyslipidemia contributes to NAFLD development [31]. Consequently, the management of NAFLD entails the correction of dyslipidemia [38].

In the present meta-analysis, our results showed that the levels of total cholesterol, LDL, and triglycerides were nonsignificantly lower in the anthocyanin group compared to the control group. Previous studies in populations other than NAFLD showed a beneficial effect of anthocyanins in reducing triglycerides [36,40,45]. A meta-analysis showed that anthocyanin intake significantly reduced serum levels of total cholesterol, triglycerides, and LDL while increasing HDL levels in patients with dyslipidemia [46]. The mechanisms of action described in the literature include the anthocyanin-induced increase in fecal excretion of neutral and acidic sterols as well as the inhibition of the synthesis of cholesterol [47]. Also, anthocyanins reduce serum apo B- and apo C-III-containing triglyceride-rich particles [48].

Overall Completeness, Applicability, and Quality of the Evidence

The included studies showed several limitations and potential bias, particularly in the domains of deviations from assigned interventions and missing outcome data. In addition, none of the studies determined the bioavailability of ingested anthocyanins by measuring the achieved serum anthocyanin levels.

There is currently no evidence to support the efficacy of anthocyanin intake in ameliorating obesity, dyslipidemia, or liver affection in NAFLD patients. Meanwhile, further studies are warranted to investigate the efficacy of higher doses for longer durations, as the results showed that the direction of pooled estimates favored anthocyanins, without reaching statistical significance. The studies showed marked heterogeneity in several outcomes, but we were unable to conduct subgroup analyses due to the small number of retrieved studies. The potential sources for the observed heterogeneity may include variations in the doses of anthocyanins, the duration of administration, and baseline variations in the studied samples. Also, stratification of patients by the grade of NAFLD allows for investigating whether mild grades may benefit from anthocyanin treatment compared to severe grades of disease.

Limitations

The included studies showed several limitations and potential bias, particularly in the domains of deviations from assigned interventions and missing outcome data. In addition, none of the studies determined the bioavailability of ingested anthocyanins by measuring the achieved serum anthocyanin levels.

## Conclusions

The results of this meta-analysis showed no evidence to support the prescription of anthocyanins for NAFLD patients for weight loss or improving dyslipidemia. However, the studies exhibited several limitations that could potentially affect the results. Future studies are warranted that should avoid the described limitations by including a larger sample size, higher doses of anthocyanins, and a longer duration of intake. Adjustment for disease severity or stratification by disease grade should be considered.

## Appendices

Appendix A

**Table 6:** Search strategy

	PubMed search terms used	Hits
#1	"non alcoholic fatty liver"[text word] OR "nonalcoholic fatty liver"[text word] OR "non alcoholic fatty liver"[text word] OR "metabolic-associated fatty liver"[text word] OR "NAFLD"[text word] OR "MAFLD"[text word] OR "Nonalcoholic Steatohepatitis"[text word] OR "non alcoholic fatty liver disease"[MeSH Terms]	44,260
#2	anthocyanin*[text word]	18,959
#3	#1 AND #2	77
#4	("Animals"[MeSH Terms] OR "Animal Experimentation"[MeSH Terms] OR "models, animal"[MeSH Terms] OR "Vertebrates"[MeSH Terms]) NOT ("Humans"[MeSH Terms] OR "Human experimentation"[MeSH Terms])	5,207,765
#5	#3 NOT #4	53

**Table 7:** Search strategy

	Cochrane Library search terms used	Hits
#1	(anthocyanin*:ti,ab,kw)	675
#2	"non alcoholic fatty liver":ti,ab,kw OR "nonalcoholic fatty liver":ti,ab,kw OR "non alcoholic fatty liver":ti,ab,kw OR "metabolic-associated fatty liver":ti,ab,kw OR NAFLD:ti,ab,kw OR MAFLD:ti,ab,kw OR "Nonalcoholic Steatohepatitis":ti,ab,kw OR [mh "non alcoholic fatty liver disease"]	5146
#3	([mh Animals] OR [mh "Animal Experimentation"] OR [mh "models, animal"] OR [mh Vertebrates]) NOT ([mh Humans] OR [mh "Human experimentation"])	3686
#4	#1 AND #2	8
#5	#4 NOT #3	8

**Table 8:** Search strategy

	WOS search terms used	Hits
#1	ALL=anthocyanin*	45,051
#2	ALL="non alcoholic fatty liver" OR ALL="nonalcoholic fatty liver" OR ALL="non alcoholic fatty liver" OR ALL="metabolic-associated fatty liver" OR ALL=NAFLD OR ALL=MAFLD OR ALL="Nonalcoholic Steatohepatitis" OR ALL="non alcoholic fatty liver disease"	56,563
#3	((ALL=anthocyanin*) AND (ALL="non alcoholic fatty liver" OR ALL="nonalcoholic fatty liver" OR ALL="non alcoholic fatty liver" OR ALL="metabolic-associated fatty liver" OR ALL=NAFLD OR ALL=MAFLD OR ALL="Nonalcoholic Steatohepatitis" OR ALL="non alcoholic fatty liver disease"))	90
#4	((ALL=anthocyanin*) AND (ALL="non alcoholic fatty liver" OR ALL="nonalcoholic fatty liver" OR ALL="non alcoholic fatty liver" OR ALL="metabolic-associated fatty liver" OR ALL=NAFLD OR ALL=MAFLD OR ALL="Nonalcoholic Steatohepatitis" OR ALL="non alcoholic fatty liver disease")) NOT ((ALL=Animals OR ALL="Animal Experimentation" OR ALL="models, animal" OR ALL=Vertebrates) NOT (ALL=Humans OR ALL="Human experimentation"))	81

**Table 9:** Search strategy

	SciELO search terms used	Hits
#1	TS=(anthocyanin*)	907
	TS=("non alcoholic fatty liver") OR TS=("nonalcoholic fatty liver") OR TS=("non alcoholic fatty liver") OR TS=("metabolic-associated fatty liver") OR TS=(NAFLD) OR TS=(MAFLD) OR TS=("Nonalcoholic Steatohepatitis") OR TS=("non alcoholic fatty liver disease")	387
	TS=(anthocyanin*) AND TS=("non alcoholic fatty liver") OR TS=("nonalcoholic fatty liver") OR TS=("non alcoholic fatty liver") OR TS=("metabolic-associated fatty liver") OR TS=(NAFLD) OR TS=(MAFLD) OR TS=("Nonalcoholic Steatohepatitis") OR TS=("non alcoholic fatty liver disease")	387
	(TS=(anthocyanin*) AND TS=("non alcoholic fatty liver") OR TS=("nonalcoholic fatty liver") OR TS=("non alcoholic fatty liver") OR TS=("metabolic-associated fatty liver") OR TS=(NAFLD) OR TS=(MAFLD) OR TS=("Nonalcoholic Steatohepatitis") OR TS=("non alcoholic fatty liver disease") ) NOT ((TS=(Animals) OR TS=("Animal Experimentation") OR TS=(Vertebrates)))	353

**Table 10:** Search strategy

	Scopus search terms used	Hits
#1	TITLE-ABS-KEY(anthocyanin*)	45,695
	TITLE-ABS-KEY ( "non alcoholic fatty liver" ) OR TITLE-ABS-KEY ( "nonalcoholic fatty liver" ) OR TITLE-ABS-KEY ( "non alcoholic fatty liver" ) OR TITLE-ABS-KEY ( "metabolic-associated fatty liver" ) OR TITLE-ABS-KEY ( nafld ) OR TITLE-ABS-KEY ( mafld ) OR TITLE-ABS-KEY ( "Nonalcoholic Steatohepatitis" ) OR INDEXTERMS ( "non alcoholic fatty liver disease" )	61,834
	(TITLE-ABS-KEY(anthocyanin*)) AND (TITLE-ABS-KEY("non alcoholic fatty liver") OR TITLE-ABS-KEY("nonalcoholic fatty liver") OR TITLE-ABS-KEY("non alcoholic fatty liver") OR TITLE-ABS-KEY("metabolic-associated fatty liver") OR TITLE-ABS-KEY(NAFLD) OR TITLE-ABS-KEY(MAFLD) OR TITLE-ABS-KEY("Nonalcoholic Steatohepatitis") OR INDEXTERMS("non alcoholic fatty liver disease"))	146
	( ( TITLE-ABS-KEY ( anthocyanin* ) ) AND ( TITLE-ABS-KEY ( "non alcoholic fatty liver" ) OR TITLE-ABS-KEY ( "nonalcoholic fatty liver" ) OR TITLE-ABS-KEY ( "non alcoholic fatty liver" ) OR TITLE-ABS-KEY ( "metabolic-associated fatty liver" ) OR TITLE-ABS-KEY ( nafld ) OR TITLE-ABS-KEY ( mafld ) OR TITLE-ABS-KEY ( "Nonalcoholic Steatohepatitis" ) OR INDEXTERMS ( "non alcoholic fatty liver disease" ) ) ) AND NOT ( ( INDEXTERMS ( animals ) OR INDEXTERMS ( "Animal Experimentation" ) OR INDEXTERMS ( "models, animal" ) OR INDEXTERMS ( vertebrates ) ) )	78
